# What’s a good prediction? Challenges in evaluating an agent’s knowledge

**DOI:** 10.1177/10597123221095880

**Published:** 2022-06-09

**Authors:** Alex Kearney, Anna J Koop, Patrick M Pilarski

**Affiliations:** 1 Department of Computing Science, 3158 University of Alberta, Edmonton, AB, Canada; 2 DeepMind, Edmonton, AB, Canada; 3 Department of Medicine, 3158 University of Alberta, Edmonton, AB, Canada; 4 Alberta Machine Intelligence Institute, Edmonton, AB, Canada

**Keywords:** Reinforcement learning, general value functions, agent knowledge

## Abstract

Constructing general knowledge by learning task-independent models of the world can help agents solve challenging problems. However, both constructing and evaluating such models remain an open challenge. The most common approaches to evaluating models is to assess their accuracy with respect to observable values. However, the prevailing reliance on estimator accuracy as a proxy for the usefulness of the knowledge has the potential to lead us astray. We demonstrate the conflict between accuracy and usefulness through a series of illustrative examples including both a thought experiment and an empirical example in Minecraft, using the General Value Function framework (GVF). Having identified challenges in assessing an agent’s knowledge, we propose an alternate evaluation approach that arises naturally in the online continual learning setting: we recommend evaluation by examining internal learning processes, specifically the relevance of a GVF’s features to the prediction task at hand. This paper contributes a first look into evaluation of predictions through their use, an integral component of predictive knowledge which is as of yet unexplored.

## 1. Introduction

A cornerstone of intelligence is knowledge. It is no surprise that much artificial intelligence research has been focused on designing algorithms that enable agents to construct knowledge of their world. In this work, we consider knowledge to be an agent’s ability to conceptualise aspects of its environment by forming predictive models of its world (Koop, 2008). The term model is sometimes restricted to estimating the probability of state transitions; however, there are many varied approaches to building world models that enable agents to better perform on decision-making tasks (Barreto et al., 2017; Ha & Schmidhuber, 2018; Jaderberg et al., 2016). In this paper, we take a broad view of what counts as a model, including predictions that forecast future input values an agent might experience. In this sense, agents construct knowledge of their world by learning to model and forecast aspects of the environment they inhabit.

The benefits of constructing knowledge by forecasting inputs are evident in computational reinforcement learning (Sutton & Barto, 2019), where an agent must learn to act optimally in order to maximize some expected cumulative future reward. Instead of finding the optimal policy directly, agents often learn the expected reward, or *value*, of states in their environment. By learning the value of a state, it becomes easier to determine what the optimal actions are.

Value functions are deeply related to the problem of control, and the distinction between the main task (finding the optimal policy) and model (estimating the value of a state) is subtle. However, modelling the environment need not end with estimating the value of states: modelling other aspects of the environment can also support decision-making (Comanici et al., 2018; Edwards et al., 2016; Jaderberg et al., 2017; Koop, 2008; Modayil et al., 2014; White, 2015). For instance, it may be useful for an agent to estimate how different inputs change in response to its behaviour (Jaderberg et al., 2017; Sherstan et al., 2020): how an agent can control what it observes through its actions. These models of the world that are independent of a particular task or goal an agent is trying to achieve can be used flexibly across different problems, including new and unseen tasks (Barreto et al., 2017; Sherstan et al., 2018).

Learning models independent of the main task not only supports agents in solving complex problems, it also forms general knowledge of the world that can be applied to new and unseen problems. How well an agent has acquired knowledge is often measured using quantitative metrics: for example, by directly measuring accuracy of a model’s estimate (Modayil et al., 2014; Pilarski & Sherstan, 2016; Sutton et al., 2011), or by examining reward received by an agent on the main task (Jaderberg et al., 2017; Schlegel et al., 2021). Systems with better quantitative outcomes are believed to better encode knowledge on a particular task.

As the main contribution of this paper, we argue that evaluating knowledge is not the same as evaluating task performance: there are new challenges that need to be addressed. In particular, a model with higher estimated accuracy does not imply that the model supports learning to solve the main problem, or task.

In what follows, we introduce this distinction by constructing two examples and related experiments: first, where traditional evaluation techniques lead to poor model choices; second, where poor model choices have down-stream consequences when used to inform decision-making. Finally, we posit that by examining internal learning processes, we can begin to evaluate agent knowledge, and show an example of how this may indeed be possible.

## 2. Background: understanding the world through general value functions

Our arguments apply broadly to evaluating machine learning models via accuracy and error alone. To focus our discussion, we ground our arguments in a single learning problem of interest: learning predictions as an agent interacts with its world. Predictions play an important role in the construction of knowledge both machines and also biological intelligence. Humans and animals continually make many predictions about their sensations (Clark, 2013; Gilbert, 2009; Nöe, 2004; Pezzulo, 2011; Pezzulo et al., 2013; Rao & Ballard, 1999; Wolpert et al., 1995). With this in mind, we use predictions to discuss the challenge of analysing knowledge in machines.

General Value Functions (GVFs) are a way for machines to learn and make predictions incrementally and online, as an agent interacts with the environment (Sutton et al., 2011). GVFs are entirely self-supervised and can be learned independent of the task an agent is undertaking through off-policy learning (Sutton et al., 2011). In this paper, we use GVFs as a computational tool to enable us to clearly make our arguments, although our arguments are independent of GVFs themselves and broadly applicable situations where models are evaluated independent of their use.

### 2.1. How GVFs are specified and learned

General Value Functions estimate the value of a signal in a sequential decision-making process. On each time-step *t*, an agent observes inputs *o*_*t*_ from the environment and takes an action *a*_*t*_ which results in a change in the environment, and thus a new observation *o*_*t*+1_. GVFs^1^ estimate the future accumulation of a *cumulant c*, where *c* is some signal of interest available to the agent through its subjective stream of experience. In the simplest case, this might be the accumulation of some element of an agent’s observation *c* ∈ *o*. The accumulation is discounted by a scalar value 0 ≤ *γ* ≤ 1 and is conditioned on a particular policy *π*: the probability of taking action *a*_*t*_ given *o*_*t*_. The discounted sum of *c*, is called the *return*, and is defined over discrete time-steps *t* as $G_{t}=E_{\pi }\left[\sum_{k=0}^{\infty }\left(\prod_{j=1}^{k}\left(\gamma_{t+j}\right)\right)C_{t+k+1}\right]$ – the expectation of how a signal will accumulate over time.

When humans interact with the environment, they construct models of the world by constantly forecasting and anticipating what will happen next (Gilbert, 2009; Rao & Ballard, 1999; Wolpert et al., 1995). Similarly, an agent can build up self-supervised models that describe the environment predictive questions such as ‘If I do this, I expect that’ with General Value Functions (Comanici et al., 2018; Ring, 2021; Sutton et al., 2011). An agent can achieve greater complexity by beginning with simple, primitive predictions about future features, and interrelating them – making forecasts of forecasts. Such primitive predictions can inform more complex predictions in two ways: one prediction may be used as an input in another; or, one prediction may be used as a cumulant *c* of another prediction. We refer to these predictions of another GVF’s output as *higher-order* predictions. By interrelating predictions, we are able to express abstract concepts that extend beyond the immediate observation stream (Koop, 2008; Schlegel et al., 2021).

**Figure 1.**
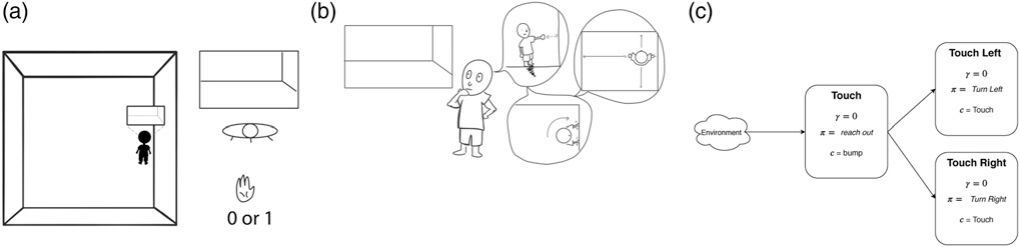
Using the limited senses available to the agent, it must construct an abstraction such that it can understand a world it can never completely see. One way of constructing an agent’s knowledge of the world is by predicting what would happen if the agent behaved a certain way. (a) Often an agent cannot observe the true state of the environment; e.g., an agent in a room may only observe what it can see in front of itself and whether the agent bumped into something. (b) Using limited sight and touch sensation, we can phrase basic spatial awareness as making predictions about moving around the room: e.g., “can I touch something in front of me?”, or “how far is the nearest wall to my left”? (c) A prediction about bumping is used toconstruct a touch prediction, the output of which is used as the target for the touch-left and touch-right predictions. Adapted from Ring (2021).

Predictions as knowledge are constructed by starting with low-level immediate predictions about sensation (Depicted in Figure 1). For example, an agent may begin to build a model of spatial awareness by predicting whether there is something in front of it: if the agent reaches out, would it be able to touch something? This simple primitive prediction could be used to inform more abstract models: for example, if the agent were to turn left or right, would there be something next to it? How far away is the nearest wall? By interrelating predictive models, we can express more abstract, conceptual aspects of the environment (Comanici et al., 2018; Koop, 2008; Ring, 1997, 2021; Singh et al., 2005; Sutton et al., 1999) (in this case, spatial awareness) in a self-supervised way.

We can estimate GVFs using Temporal-difference (TD) learning (Sutton, 1988). In TD learning, we estimate a value-function *v* such that $v\left(\phi \left(o_{t}\right)\right)\approx E_{\pi }\left[G_{t}|o_{t}\right]$ : we learn a function that estimates the return at a given time-step given the agent’s observations. On each time-step, the agent receives a vector of observations $o\in {\mathbb{R}}^{m}$ . A function approximator $\phi :o\to {\mathbb{R}}^{n}$ – such as a neural net, Kanerva coder, or tile coder – encodes observations into a *feature vector*. The estimate for each time-step *v* (*ϕ*(*o*_*t*_)) function of learned weights $w\in {\mathbb{R}}^{n}$ , and the current feature vector – *v* (*o*_*t*_) = *w*^*⊤*^*ϕ*(*o*_*t*_).

We call the parameters of the learning methods *learning parameters*. Learning parameters change how the value function is approximated, but do not change what the value function is about. Learning parameters include the step-size also know as learning rate, *α* which scales updates to the weights, the eligibility trace decay *λ* and the function approximator *ϕ* used to construct state.

### 2.2. The challenge of constructing knowledge

One challenge for constructing models of the world is deciding of all the predictions an agent could learn to make, which subset can inform decision-making best. That is, an agent must choose from all the possible predictions which it could make, the subset of predictions that will help it achieve its goals. Not all predictions are created equally: two approximate GVFs may have the same question parameters -*γ*, *π*, and *c* - and yet produce very different estimates. Disparity in accuracy can be caused many factors including: the learning parameters chosen, the distribution of experience trained on, feature construction, and the step-size parameter. Each factor contributes to the how well an estimator can be learned. To be able to compare estimators, we must have some metric or means of evaluating them.

In this manuscript, we demonstrate how strict measures of model accuracy can be misleading in assessing an agent's knowledge of the world. We argue this over two experiments. In the first experiment, we demonstrate how common online evaluation techniques can be misleading when choosing between two models of the same aspect of an environment. Selecting between two identically specified models is the most primitive choice an agent must make when constructing predictive knowledge: a choice that is surprisingly not straightforward. In the second experiment we demonstrate how relying on such evaluation metrics undermine an agent’s ability to reason about its environment --particularly as the agent relies on these estimates to further develop abstract conceptualisations of its world. This presents a further challenge, as the motivation of constructing knowledge is its application in decision-making and reasoning. Having brought to light these two core challenges, we then develop a method of assessing predictive models of the world, providing a path to alleviating these barriers to evaluating an agent's knowledge of the world.

## 3. Experiment 1: how poor evaluation impacts predictive features

In this section we construct and example where traditional evaluation techniques lead to poor model choices. To do so, we construct a synthetic prediction problem and explore how a common online error metrics can be misleading.

### 3.1. Evaluation by empirical return error

To choose between models, we need to have a method of comparing them. We cannot compare GVFs to the true expected return of their cumulant *c*: we do not have access to the true return from the stream of data available to an agent. Instead, we often assess a GVF’s accuracy based on an estimate of the true return, the *empirical return error*: the difference between the current estimate *v* (*ϕ*(*o*_*t*_)) with an approximation of the true return (Edwards et al., 2016; Günther et al., 2016; Pilarski et al., 2012). The return is estimated by maintaining a buffer of length *b* of previous cumulants *c*, such that $\tilde{G_{t}}=\sum_{k=0}^{b}\left(\prod_{j=1}^{k}\left(\gamma_{t+j}\right)\right)C_{t+k+1})$ . We may then construct an error for time-step *t* given the agent’s experience by $\tilde{G_{t}}-V_{t}(\phi \left(o_{t}\right)$ . The empirical return error is not objective. Note, it depends on what the agent happens to experience – it can only express the error for observations represented in the buffer. It does not capture error for all possible observations or states of the world.

In simple Markov Reward Processes, this may not be an issue: maintaining a large enough buffer *b* will yield an error relatively unbiased over states. However, in many domains of interest, this is not possible: that is, in robotics the state-space is often so immense that maintaining a buffer of observations would be a time-intensive and impractical demand. Instead, applications often settle for an empirical return error that covers only a portion of the state-space (Edwards et al., 2016; Günther et al., 2016; 2020; Pilarski & Sherstan, 2016). In doing so, some states are inherently prioritized over others, as they are gerrymandered into two categories: the portions of state-space that are evaluated, and the portions that are not. When evaluating methods in this way, it is implicit that some of the states are privileged over others: that error matters more in one set of states over another (Sutton et al., 2009).

### 3.2. A synthetic example

**Figure 2.**
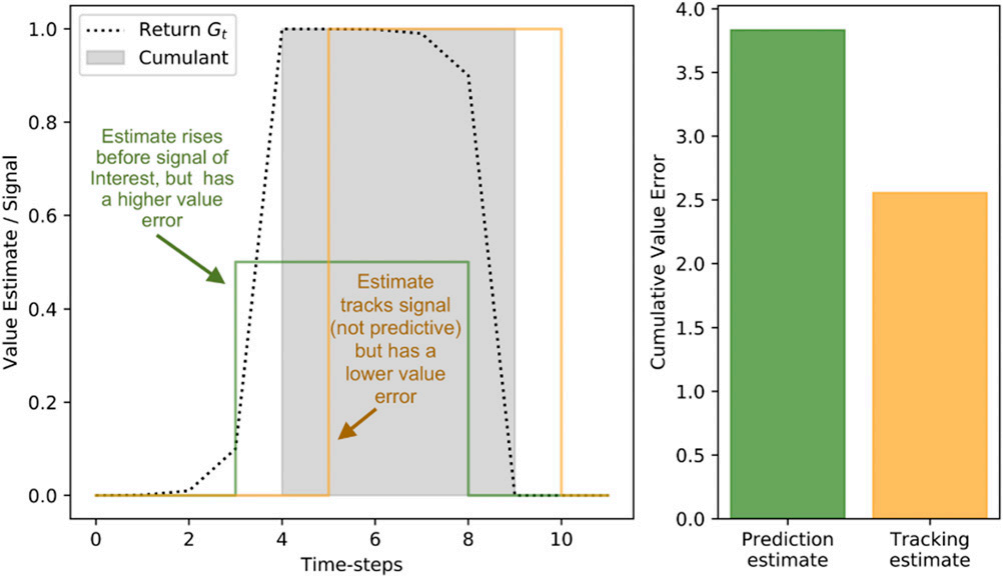
Two estimates of the same signal: one in green and one in orange. The cumulant *c* is indicated by the grey square pulse. The return of *G*_*t*_ of the cumulant is presented as a dotted line. Two hypothetical estimates of the return are presented in green and orange.

We present two hypothetical estimators of the same value-function in Figure 2 as an example of how empirical error can be gerrymandered by state. A binary square pulse is the cumulant *c* for which two hypothetical value functions estimate the discounted return. The dotted line is the scaled return *G*_*t*_ of the cumulant *c* with a discount factor of *γ* = 0.3 that is being estimated. A perfect prediction will match the return *G* of the signal: rising before the signal of interest *c* rises, and falling before the pulse returns to 0. Such a value estimate is predictive – it forecasts the signal of interest.

Two hypothetical value functions are presented: (1) In orange, an estimator that tracks the cumulant by returning the last observed cumulant value; (2) in green, an imperfect but predictive estimate. The tracking estimator is not predictive: it rises and falls after the signal of interest. The predictive estimate does not exactly match the return being estimated, but rises and falls prior to changes in the underlying cumulant being estimated. While the tracking estimate fails to anticipate the square pulse, it has a lower empirical return error for the time period presented. If we were evaluating the two predictions and choosing between these two estimators using prediction error alone, we would be led to believe that the tracking estimator is superior to the predictive estimator: it has a lower cumulative error. This becomes an issue when these estimates are intended to inform decision-making. For instance, if an agent is predicting a collision, identifying the collision has occurred after the fact is not useful in supporting decision-making.

### 3.3. Experimental summary

While this synthetic example is contrived, there are many situations in which we would want to make such a prediction; being able to detect the onset of events is often useful in decision-making (Modayil & Sutton, 2014; Ring, 2021; Schlegel et al., 2021). For example, in the previous section, we worked out an example where an agent built a sense of spatial awareness (Figure 1) by predicting whether it could touch something in front of itself; In the spatial awareness example, touch is a binary signal that rises and falls, similar to this simple synthetic example. Such predictions are not made in a vacuum: the motivation for learning models is to use them to inform decision-making.

## 4. Experiment 2: how performance is impacted by poor predictive features

With a simple example, we demonstrated how accuracy can be misleading in differentiating between forecasts. Such forecasts are motivated by their use: using the learned estimates as either (1) predictive input features to another learning process, or (2) a signal of interest for further abstract predictions. We now discuss how dependence on accuracy negatively impacts down-stream learning processes that use these learned estimates, and can critically undermine representation learning. To this end, we construct a network of interrelated predictions: a collection of predictions where a learned estimate is used to inform other learning processes.

The core motivation of learning models of the environment is to use such models to improve decision-making. The appeal of learning GVFs is the ability to build modular, and hierarchical forecasts about the world – forecasts that can be used as predictive features for other learning processes. This is achieved by (1) using an estimate as an input feature when making a higher-order GVF, or (2) using a learned estimate as a cumulant for another GVF. In this section, we demonstrate that poor evaluation in lower-order GVFs has consequences for the performance of higher-order GVFs. In order to demonstrate these challenges in evaluation, we turn our attention to the off-policy prediction setting.

### 4.1. Estimating error for off-policy learning

Off-policy predictions are conditioned on a particular behaviour. While conditioned on a specific policy, off-policy GVFs can be learned while engaging in behaviours that do not strictly match the target policies of the prediction. Because the behaviour an agent is engaging in may not precisely match the policy an off-policy prediction is on, we cannot accurately compute the empirical return error (Sutton et al., 2009). The buffer *b* collected from the agent’s experience may represent experience induced by a policy different from the policy which a prediction is specified; therefore, the return calculated from the buffer will not be representative of the off-policy return.

An off-policy error metric which can be calculated incrementally online, is RUPEE: the Recent Unsigned Projected Error Estimate (White, 2015). RUPEE estimates the mean squared projected Bellman error of a single GVF.^2^ Intuitively, RUPEE is an estimate of learning progress with respect to the input features used by the agent in learning. While RUPEE does not imply prediction accuracy, RUPEE provides a computationally efficient way to determine when a forecast learned off-policy is approaching its best estimate (White, 2015).

RUPEE requires an additional parameter *β*_0_ > 0 which specifies a decay rate for the exponential moving average of both *τ* and $\bar{\delta e}$ – an exponential moving average of the TD error and eligibility traces.^3^ A higher *β* value results in a longer horizon for the moving average. Where *e* is the forecast’s eligibility traces, *δ* is the TD error, and *h* is the same as the update in GTD (*λ*); RUPEE is estimated as follows

$$\tau \leftarrow \left(1-\beta_{0}\right)\tau +\beta_{0}$$

$$\beta \leftarrow \frac{\beta_{0}}{\tau }$$

$$\bar{\delta e}\leftarrow \left(1-\beta \right)\bar{\delta e}+\beta \delta e$$

$$\text{RUPEE}\leftarrow \sqrt{\left|{\hat{h}}^{⊤}\bar{\delta e^{\beta }}\right|}$$

As was the case when evaluating on-policy predictions via empirical return error, by estimating off-policy learning progress using RUPEE, we are unable to differentiate between useful and useless estimators.

### 4.2. Predictions estimated

In the previous experiment we demonstrated how using prediction error as a direct proxy for model quality can mislead. We now demonstrate how mis-evaluating the quality of GVFs can lead to poor performance in general. To do so, we introduce a network of predictions adapted from Ring’s thought experiment on spatial knowledge (Ring, 2021), depicted in Figure 2. In this setting, the most basic GVF is touch: in plain terms, predict whether the agent would feel a surface if it extended its hand. Two natural higher-order predictions can be based on this: touch-left and touch-right (predict whether the touch GVF would activate if the agent turned left or right, respectively). Further higher-order predictions can build up to basic navigation and spatial awareness (Ring, 2021). However, in order to successfully build these concepts, we must first get the simple, primary prediction right.

### 4.3. Experimental environment

**Figure 3.**
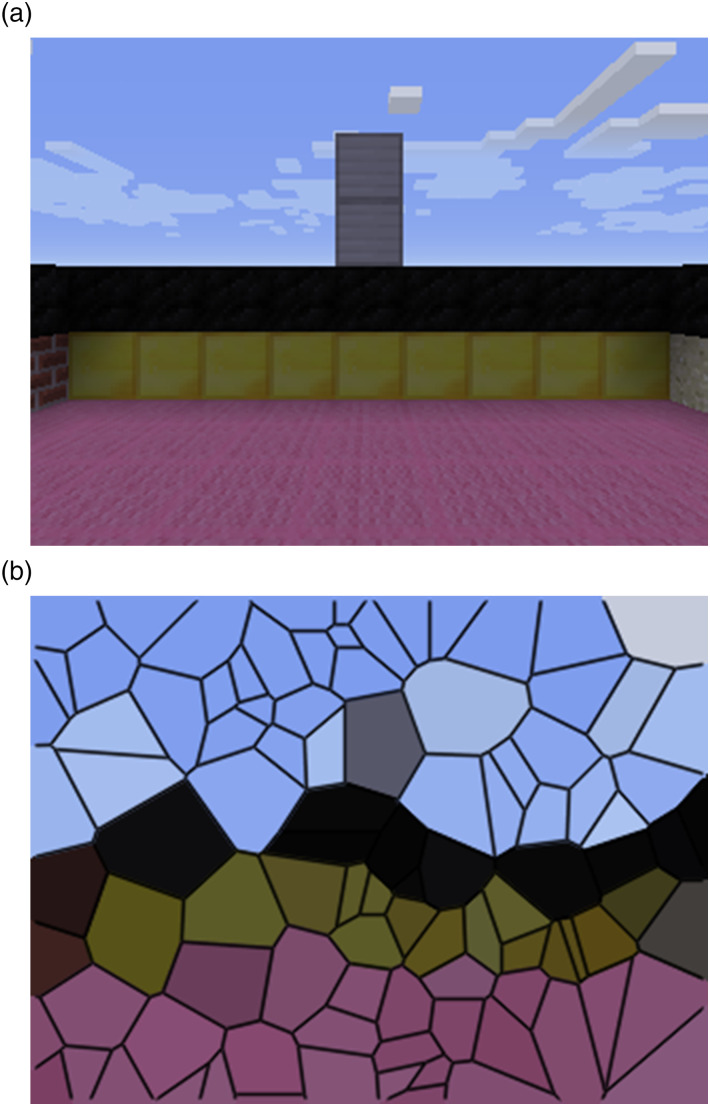
A visual representation of our agent approximating the visual input by sub-sampling 100 random pixels. (a) The visual input the agent totaling 320×480 pixels (b) Visualization of the image subsampled to 100 random pixels.

These predictions are made in a MineCraft (Johnson et al., 2016) grid-world that reflects the spatial awareness task we previously introduced (Figure 2).^4^ The world is a square pen which is 30 × 30 and two blocks high. The mid-section of each wall has a silver column, and the base of each wall is a unique colour. On every time-step, the agent receives observations *o*_*t*_ which contain: (1) the pixel input from the environment (Figure 3(a)), and (2) whether or not the agent is touching something.

## 5. Results

Similar to the previous synthetic example, we have two sets of value functions: one that predicts, and one that tracks. We construct two GVF networks that are specified with the same question parameters, but differ in answer parameters used. Both sets of GVFs are approximating the same values; however, the way they learn their approximation differs. One touch prediction uses a Tile Coder (Sherstov & Stone, 2005; Sutton & Barto, 2019) as a function approximator, and the tracking GVF uses only a single bias bit as a representation. We choose this representation, as it is clear that a bias bit is insufficient to inform any of the chosen predictions: we cannot predict whether the agent can touch a wall using a single bit to represent our MineCraft world.

**Figure 4.**
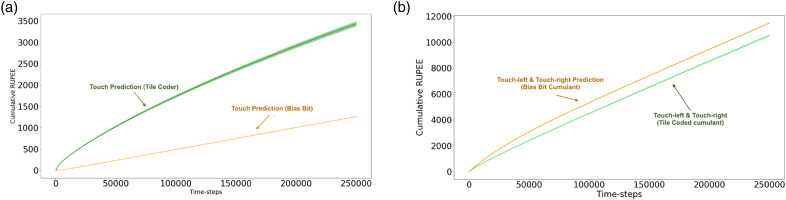
Cumulative Recent Unsigned Projected Error Estimate (RUPEE over 250,000 time-steps for the ‘touch-left’ and ‘touch-right’ predictions averaged over 30 independent trials. (a) Cumulative RUPEE for tile-coded touch estimate (green) and bias-bit touch estimate (orange).The tracking estimate accumulates error at a slower rate than the anticipatory prediction. Evaluating based on RUPEE alone, we would be led to believe that the tracking model is best, despite leading to catastrophic prediction error when used to inform touch-left and touch-right (c.f. Figure 5). The anticipatory touch estimate has a greater accumulation of error throughout the experiment despite being a better estimator for informing touch-left and touch-right predictions (b) Cumulative RUPEE for touch-left and touch-right estimates which use as a cumulant the tile-coded (green) and bias bit (orange) touch estimate. Estimates dependent on the tracking GVF for learning have a greater cumulative error than the GVFs dependent on the Tile Coder GVF. Error as accumulated at roughly the same rate as the anticipatory GVFs, making it challenging to distinguish which of the prediction is better, despite wildly different outcomes when comparing prediction to ground-truth (c.f. Figure 5). The error of the lower-order models does not always determine their effectiveness in informing further learning.

**Figure 5.**
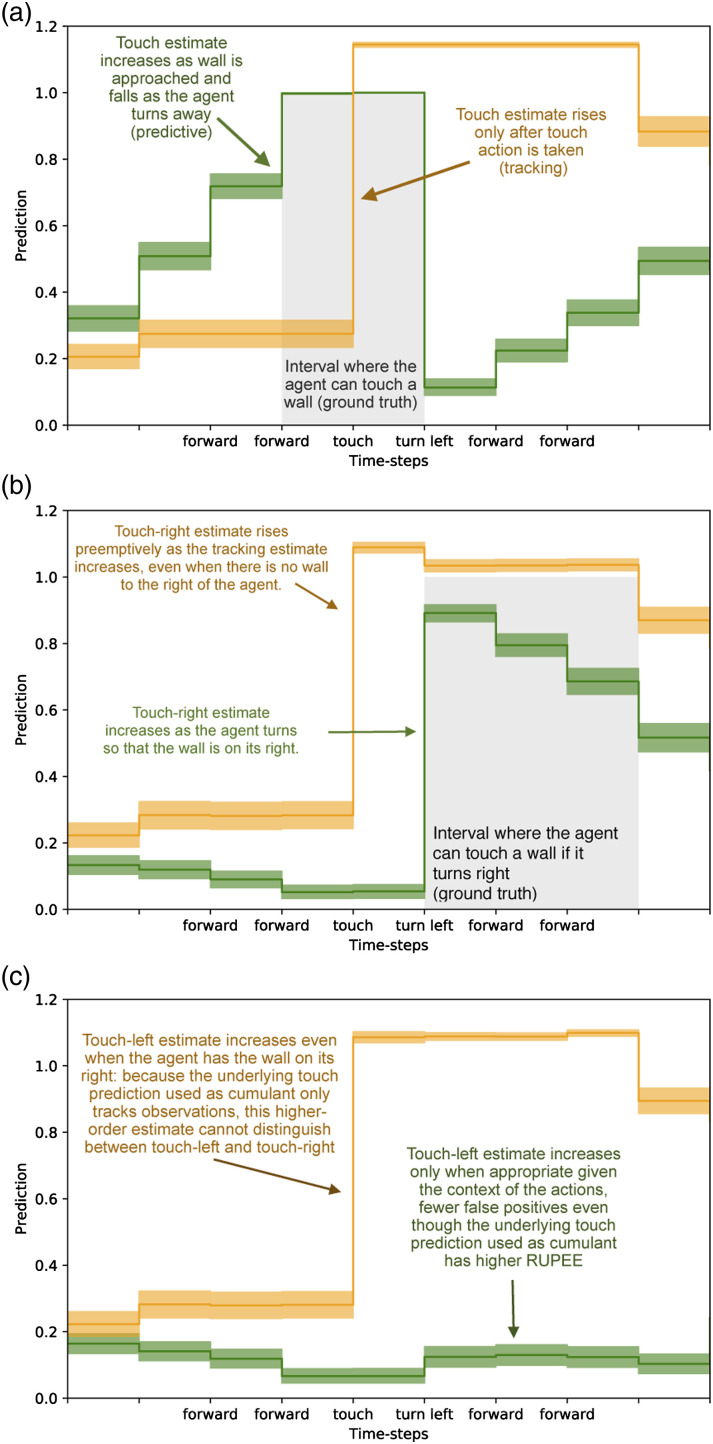
Each sub-figure depicts estimates of each of the GVFs in our networks for 150 examples of the agent approaching a wall and then turning left. Five examples of the trajectory are drawn from 30 independent trials: results presented are averaged over 150 examples of the same trajectory. (a) Tile-coded touch estimate (green) and bias-bit touch estimate (orange) (b) touch-right estimates which use as a cumulant the tile-coded (green) and bias bit (orange) touch estimate (c) touch-left estimates which use as a cumulant the tile-coded (green) and bias bit (orange) touch estimate.

This experimental setup directly parallels our on-policy synthetic example in a more complex environment. As was the case in the previous thought experiment, by comparing the two touch predictions based on their error (Figure 4(a)), we would be lead to conclude that the bias bit GVF is superior to the tile-coded GVF – we would conclude that the prediction that does not predict is superior. When we examine the actual predictions made by each GVF, we see that the predictive estimate with a greater RUPEE more closely anticipates the signal of interest (Figure 5(a)). The reason why the bias bit prediction is poor is because it tracks. An architect designing a system understands this prediction is poor because it is redundant: the immediate sensation of touch tells us whether or not an agent *is* touching something. The intent of the prediction is to compactly express whether or not an agent *can* touch a wall without needing to engage in the behaviour. When the agent does touch a wall, the prediction is updated and stored in the weights of the GVF. Only when the agent is touching a wall will the bias bit GVF predict that it can touch a wall. By looking at RUPEE alone, we miss this critical shortcoming.

These predictions are not learned in a vacuum: the purpose of making the touch prediction, is to enable the higher-order predictions to be learned. In systems that use GVFs to construct an agent’s knowledge of the world, these predictions are intended to inform further learning processes: either other value functions that describe more abstract aspects of the world, or the behaviours an agent uses to accomplish its goals. Low RUPEE or low return error in an estimator does not necessarily equate to more useful predictions for these further decision-making purposes. The challenges of differentiating between a good and bad touch prediction have an impact which extends beyond the single prediction and influences the touch-left and touch-right predictions.

We want not only an accurate touch prediction, but one which is capable of informing Touch-Left and Touch-Right predictions. In Figure 5, we display the RUPEE of Touch-Left and Touch-Right. There are two sets of these predictions: the first, using the bias bit GVF’s prediction as its cumulant; the second, using the tile-coded GVF as its cumulant. In this layer, the GVFs all share the same function approximator: they both use sufficient representations to learn a reasonable estimate. In this case, a random sub-sampling of the pixel input, binary touch signal, and touch prediction are all tiled together to construct the state for each GVF. The only differentiating factor is which cumulant is used: the prediction from either the tracking touch GVF, or the anticipatory touch GVF.

When we examined the first layer’s Touch predictions, the tracking GVF seemed superior based on RUPEE. When we examine the RUPEE of the second set of predictions (Figure 4(b)), we catch a glimpse of the down-stream effects of this misunderstanding. Although only slight, the GVFs dependent on the tracking Touch prediction have a higher RUPEE than those using the predictive Touch GVF. This point is brought into focus when we examine the predictions made by each touch-left and touch-right prediction (Figures 5(b) and 5(c)). When we examine average trajectories where the agent approaches a wall and turns left, the touch-right prediction using the tracking touch GVF as a cumulant (Figure 5(b), in orange) rises and falls with its underlying GVF. The touch-right prediction with a tracking cumulant predicts wall even before turning such that the wall is to its right, while the touch-right prediction with a predictive cumulant is able to better match the ground-truth. This disparity is further exacerbated in Figure 5(c), where we see that the touch-left prediction dependent on the tracking touch GVF as a cumulant incorrectly anticipates a wall is on its left, even as it turns away from it. Through examining the error – the metric used to inform predictive knowledge architectures – we miss this. The use of a prediction tells us more about the quality of that prediction than error alone. By using a poor underlying touch prediction, the higher-order GVFs become un-learnable.

### 5.1. Experimental summary

We demonstrated that poor behaviour of estimates can be hidden by commonly used error metrics. This kind of inquiry into the structure of predictions is not easily automated: it relies on inspection by system designers. Moreover, these precise comparison are limited to simple domains. The room our agent inhabits is so simple that we can acquire the ground-truth in order to examine the predictions as is done in Figure 5. In many domains of interest, this ease of comparison is simply impossible. Each of these factors further frustrates the problem of determining what to learn, and whether particular GVFs are useful for informing decision-making.

There is no metric the automate the analysis of prediction usefulness for decision-making. Consequently, system designers rely on the metrics currently available: namely, prediction error; designers are missing metrics that summarize and evaluate the usefulness of features. In the following section, we propose a metric to fill this gap: evaluation of prediction usefulness by examining a value function’s internal learning process.

## 6. Proposal: evaluate feature relevance

We demonstrated that error in isolation of any additional information is misleading: empirical return error and RUPEE are insufficient to determine whether a model is useful for informing down-stream decision-making by an agent. This inability to assess the usefulness of predictions is a major hurdle, the purpose of constructing knowledge is its use in supporting decision-making. If measures of accuracy verified using data available to the agent are not enough to assess the usefulness of a model, what should a designer do?

We need not only look at signals external to the agent for clues about performance: we can also look inwards and examine the learning process to assess an agent’s knowledge – how the agent is modifying its parameters. Examining an agent’s parameters is not unusual. For example, Unexpected Demon Error (UDE), can be used to gauge how ‘surprising’ a given observation is to an agent (White, 2015). By examining the surprise, we can gauge how current experience relates to past experiences – for example, detecting faults in a system (Günther et al., 2018).

Similarly, there are many such parameters that an agent can modify during learning, and that modification can be monitored. Of particular interest are meta-learning methods: higher-order learning processes that modify the learning parameters of an agent (e.g. IDBD (Sutton, 1992)). One notable example is step-size (learning rate) adaptation.

More broadly, we can view these forms of step-size adaptation as the most basic form of *representation learning*. Representation learning describes (Bengio et al., 2013) how an agent encodes data or experience in order to support decision-making. By assigning each individual input a specific step-size, an input is weighted proportional to its relevance to some down-stream learning task. For instance, TD Incremental Delta-Bar-Delta (TIDBD) (Kearney et al., 2019) assigns a step-size *α*_*i*_ to each weight *w*_*i*_, adjusting the step-size based on the correlation of recent weight updates. If many weight updates are made in the same direction, then a more efficient use of data would have been to make one large update with a larger *α*_*i*_. If an update has over-shot, then the weight updates will be uncorrelated, and thus the step-size should be smaller.

All else being equal, a good model is one whose features are well aligned with the prediction problem at hand. Even in early-learning where an agent is adjusting its model, or in situations where non-stationarity in the environment may introduce unexpected error, if the features are relevant to the prediction task we can expect a reasonable model to be learned. One way to determine the relevance of features is by learning step-sizes.

### 6.1. Derivation of off-policy TIDBD

To demonstrate how step-sizes as feature relevance can be informative, we generalize TIDBD (Kearney et al., 2019) to GTD (*λ*), creating a step-size adaptation method suited for the off-policy touch, touch-left, and touch-right predictions we previously introduced. Off-policy AutoStep for GTD adds a few additional memory parameters to perform step-size adaptation.

Here, we derive the relevant updates as follows. TIDBD minimizes *δ*^2^ the squared TD error with respect to meta-weights *β* that specify the agent’s step-size on each time-step

(1) $$\begin{array}{l} \beta_{i,t+1}=\beta_{i,t}-\frac{1}{2}\theta \frac{\partial \delta_{t}^{2}}{\partial \beta_{i}}\\ \;\;\;\;\;\;\;\;\;\;\;\;=\beta_{i,t}-\frac{1}{2}\theta \sum_{j}\frac{\partial \delta_{t}^{2}}{\partial w_{j,t}}\frac{\partial w_{j}}{\partial \beta_{i}} \end{array}$$

We expand $\frac{\partial \delta_{t}^{2}}{\partial \beta_{i}}$ using the chain-rule. As in (Sutton, 1992), we make the assumption that the effect of changing the step size *α*_*i*_ = exp (*β*_*i*_) for some feature ϕi,t will predominantly be on the weight *w*_*i*_

(2) $$\beta_{i,t+1}\approx \beta_{i,t}-\frac{1}{2}\theta \frac{\partial \delta_{t}^{2}}{\partial w_{i,t}}\frac{\partial w_{i,t}}{\partial \beta_{i}}$$

We are minimizing the TD error *δ* = *c*_*t*+1_ + *γV* (*ϕ*_*t*+1_ − *V*(*ϕ*)), where *c* is the cumulant, *γ* is the discount factor, and *V* is our value function, and *ϕ* is the state as constructed by a function approximator. Given *δ* is a biased estimate of the error, dependent on our value function *V*, we take the semi-gradient − *V* (*ϕ*_*t*_)

(3) $$\begin{array}{l} -\frac{1}{2}\frac{\partial \delta_{t}^{2}}{\partial w_{i,t}}=-\delta \frac{\partial \left[-V\left(\phi_{i,t}\right)\right]}{\partial w_{i,t}}\\ \;\;\;\;\;\;\;\;\;\;\;\;\;\;\;\;\;\;\;\;=\delta_{t}\phi_{i,t} \end{array}$$

(4) $$\beta_{i,t+1}\approx \beta_{i,t}+\delta_{t}\phi_{i,t}\frac{\partial w_{i,t}}{\partial \beta_{i}}$$

We then describe $\frac{\partial w_{i,t}}{\partial \beta_{i}}$ as *ω*. GTD(*λ*) updates the weights as *w* ← *w* + *α*[*δe* − *γ*(1 − *λ*) (*e*^*⊤*^*h*)*ϕ*_*t*+1_]. We can then write the update to *ω* recursively as follows

(5) $$\begin{array}{l} \omega_{t+1}=\frac{\partial }{\partial \beta }\left[w+\alpha \left(\delta e-\gamma \left(1-\lambda \right)\phi_{t+1}e_{t}^{⊤}h_{t}\right)\right]\\ \;\;\;\;\;\;\;\;\;\;=\omega_{t}+\alpha \delta e+\alpha e\frac{\partial }{\partial \beta }\left[\delta \right]+\alpha \delta \frac{\partial }{\partial \beta }\left[e\right]\\ \;\;\;\;\;\;\;\;\;\;\;-\alpha \gamma \left(1-\lambda \right)\phi_{t+1}e^{⊤}h-\alpha \gamma \left(1-\lambda \right)\phi_{t+1}\frac{\partial }{\partial \beta }\left[e^{⊤}h\right]\\ \;\;\;\;\;\;\;\;\;\;\;\approx \omega_{t}+\alpha \delta e-\alpha \omega_{t}\phi_{t}e-\alpha \gamma \left(1-\lambda \right)\phi_{t+1}e^{⊤}h\\ \;\;\;\;\;\;\;\;\;\;\;-\alpha \gamma \left(1-\lambda \right)\phi_{t+1}e^{⊤}\frac{\partial }{\partial \beta }\left[h\right]\\ \;\;\;\;\;\;\;\;\;\;\;=\omega_{t}+\alpha \left(\delta e-\omega_{t}\phi_{t}e-\gamma \left(1-\lambda \right)\phi_{t+1}\left(e^{⊤}h+e^{⊤}\eta_{t}\right)\right) \end{array}$$

In GTD (*λ*), the bias-correction updated update is *h* ← *h* + *α*(*δe* − (*h*^*⊤*^*ϕ*_*t*_)*ϕ*_*t*_). Similar to *ω*, we define $\frac{\partial h_{t}}{\partial \beta }$ as *η*. The *η* update is as follows

(6) $$\begin{array}{l} \eta_{t+1}=\frac{\partial }{\partial \beta }\left[h_{t}+\alpha \left(\delta e-\left(h^{⊤}\phi_{t}\right)\phi_{t}\right)\right]\\ \;\;\;\;\;\;\;\;=\eta_{t}+\alpha \delta e+\alpha \frac{\partial }{\partial \beta }\left[\delta \right]e+\alpha \delta \frac{\partial }{\partial \beta }\left[e\right]-\alpha \left(h^{⊤}\phi_{t}\right)\phi_{t}\\ \;\;\;\;\;\;\;-\alpha \frac{\partial }{\partial \beta }\left(h^{⊤}\phi_{t}\right)\phi_{t}\\ \;\;\;\;\;\;\;\approx \eta_{t}+\alpha \delta e-\alpha \omega_{t}\phi_{t}e-\alpha \left(h^{⊤}\phi_{t}\right)\phi_{t}-\alpha \left(h^{⊤}\phi_{t}\right)\phi_{t} \end{array}$$

We now have our three additional updates defined for GTD (*λ*) IDBD. This results in our GTD IDBD. We now have all the features for a GTD version of IDBD

(7) $$\beta \leftarrow \beta +\theta \delta \phi_{t}\omega_{t}$$

(8) $$\eta \leftarrow \eta +\alpha \left(\left(e\left(\delta -\omega_{t}\right)-{\left(h+\eta \right)}^{⊤}\phi_{t}\right)\phi_{t}\right)$$

(9) $$\begin{array}{c} \omega \leftarrow \omega +\alpha \left(e\left(\delta -\omega_{t}\phi_{t}\right)-\gamma \phi_{t+1}\left(1-\lambda \right)e^{⊤}\left(h+\eta \right)\right) \end{array}$$

To generalize AutoStep (Mahmood et al., 2012) to GTD(*λ*), we need two more additions to GTD(*λ*): (1) a running average of meta-weight updates to prevent instability in our meta-weights caused by dramatic changes in the target of the underlying learning method, and (2) a normalization by the *effective step size* to prevent over-shooting on an individual example.

The effective step size describes the amount by which the error has been reduced on a particular example after a weight update. If the effective step-size is greater than one, then we have over-shot on a particular example. To prevent over-shooting, we divide the step-size on each time-step by $\max\left(1,\frac{\delta_{t}\left(t\right)-\delta_{t+1}\left(t\right)}{\delta_{t}\left(t\right)}\right)$ To find the effective step-size, we simplify the following

(10) $$\begin{array}{l} \frac{\delta_{t}\left(t\right)-\delta_{t+1}\left(t\right)}{\delta_{t}\left(t\right)}=\frac{1}{\delta_{t}\left(t\right)}-[\left(C_{t+1}+\gamma V_{t}\left(\phi_{t+1}\right)-V_{t}\left(\phi_{t}\right)\right)\left(C_{t+1}+\gamma V_{t+1}\left(\phi_{t+1}\right)-V_{t+1}\left(\phi_{t}\right)\right)]\\ \;\;\;\;\;\;\;\;\;\;\;\;\;\;\;\;\;\;\;\;\;\;\;\;\;\;\;\;\;\;=\frac{1}{\delta_{t}\left(t\right)}[\left(\gamma V_{t}\left(\phi_{t+1}\right)-V_{t}\left(\phi \left(t\right)\right)\right)-\left(\gamma V_{t+1}\left(\phi_{t+1}\right)-V_{t+1}\left(\phi_{t}\right)\right)] \end{array}$$

We simplify to the resulting effective step size

(11) $$\begin{array}{c} {\left[\alpha e-\frac{\gamma \left(1-\lambda \right)\phi_{t+1}e^{⊤}h}{\delta }\right]}^{⊤}\left[\phi_{t}-\gamma \phi_{t+1}\right] \end{array}$$

Having found the effective step-size, we must define an update normalizer. On each time-step, IDBD updates the step-sizes by *δϕω*. We take a decaying trace of the maximum weight update, $\xi \leftarrow \max\left(|\delta \phi \omega |,\xi +\frac{1}{\tau }\alpha \phi e\left(|\delta \phi \omega |-\xi \right)\right)$ , where *τ* is a parameter that specifies how quickly *ξ* decays. This has the effect of maintaining a decaying trace of the maximum update such that a large change in the underlying learning target does not lead to instability in the step-size parameter update.

Algorithm 1 GTD (*λ*) with AutoStep step-size tuning.

1: **Initialise:**

2: Initialize vectors *ω*, *η*, *α*, *e*, *ξ*, and *w* of size *n* (number of features).

3: Set *τ* as a decay value, for example, 10^4^ and *θ* as a meta step-size (e.g. 10^−2^).

4: **begin:**

5: Observe initial state *ϕ*

6: Take initial action *a*

7: **repeat** interaction with environment:

8: Observe next state *ϕ*^′^ and cumulant *c*

9: *δ* ← *c* + *γw*^*⊤*^*φ*^′^ − *w*^*⊤*^*φ*_*t*_

10: *ξ* ← **max**:

|*δφω*|,

$\xi +\frac{1}{\tau }\alpha \phi e\left(|\delta \phi \omega |-\xi \right)$

11: **for***i* = 1, 2, …, *n*: **do**

12: **if***ξ*_*i*_ ≠ 0: **then**

13: $\alpha_{i}\leftarrow \alpha_{i}\exp\left(\theta \frac{\delta \phi \omega }{\xi_{i}}\right)$

14: **end if**

15: **end for**

16: *M* ← **max**:

1,

${\left[\alpha e-\frac{\gamma \left(1-\lambda \right)\phi_{t+1}e^{⊤}h}{\delta }\right]}^{⊤}\left[\phi_{t}-\gamma ,\phi_{t+1}\right]$

17: $\alpha \leftarrow \frac{\alpha }{M}$

18: $\rho \leftarrow \frac{\pi \left(\phi ,a\right)}{\mu \left(\phi ,a\right)}$

19: *w* ← *w* + *α*(*δe* − *γ*(1 − *λ*)*e*^*⊤*^*hϕ*^′^)

20: *h* ← *h* + *α*(*δe* − (*h*^*⊤*^*φ*)*φ*)

21: *e* ← *ρ*(*eγλ* + *φ*)

22: $\omega \leftarrow \omega +\alpha \left(e\left(\delta -\omega \phi \right)-\gamma \phi^{\prime }\left(1-\lambda \right)e^{⊤}\left(h+\eta \right)\right)$

23: $\eta \leftarrow \eta +\alpha \left(\left(e\left(\delta -\omega_{t}\right)-{\left(h+\eta \right)}^{⊤}\phi \right)\phi \right)$

24: *φ* ← *φ*^′^

25: **until** termination

These updates can then be combined with the underlying GTD(*λ*) updates to produce and Autostep GTD (*λ*) (Algorithm 1).

## 7. Experiment 3: analysing feature relevance

### 7.1. Experimental Setup

**Figure 6.**
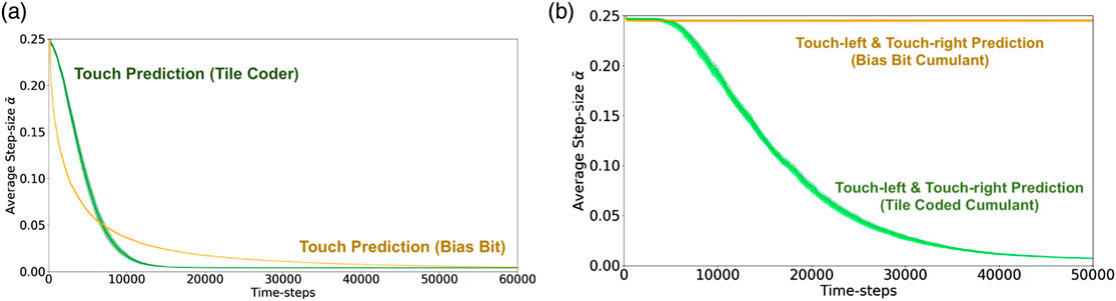
The average active step-sizes for each layer of both the prediction and tracking networks averaged over 30 independent trials. Error bars are standard error of the mean. (a) The average active step-size for both touch predictions. Anticipatory prediction in green; tracking based prediction in orange. (b) Average active step-size for the touch-left and touch-right predictions. Anticipatory predictions in green; tracking-based predictions in orange.

Having generalized TIDBD to GTD (*λ*), we now return to the MineCraft domain and perform the same experiments, now using step-size adaptation. In Figure 6, the average active^5^ step-size value for the duration of the experiment is depicted. As was the case in the prior experiments, we have two agents each learning three predictions: touch, touch-right and touch-left. One agent has a representation sufficient to learn the underlying touch prediction with reasonable accuracy (green), while the other does not (orange).

### 7.2. Results: examining feature relevance

**Figure 7.**
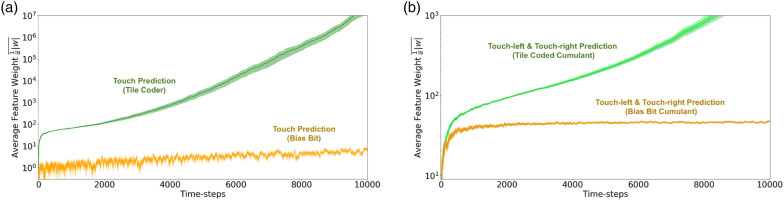
The average weighted feature relevance $\bar{\frac{1}{\alpha }|w|}$ for each layer of both the prediction and tracking networks. Each is run over 30 independent trials. Error bars are standard error of the mean. (a) Average weighted feature relevance $\bar{\frac{1}{\alpha }|w|}$ for touch predictions. (b) Average weighted feature relevance $\bar{\frac{1}{\alpha }|w|}$ for the touch-left and touch-right predictions. Anticipatory predictions in green; tracking-based predictions in orange.

By examining the step-size values, we are able to discriminate between the tracking and predictive touch-left and touch-right predictions (Figure 6(b)); however, we find that the tracking and predictive touch predictions are not appreciably different when examining their step sizes late in learning progress (as shown in Figure 6(a)). Independent of learned weights, step-sizes do not tell the full story; our step-sizes *α* are a weighting of our features *ϕ* when learning some weights *w*. The learned step-sizes *α* in combination with the learned weights *w* give us greater insight into the performance of our GVFs. In Figure 7, a combination of the absolute value of the learned weights and step-sizes are plotted: $\bar{\frac{1}{\alpha }|w|}$ . We take $\frac{1}{\alpha }$ , as the magnitude of the step-size describes progress in learning. Intuitively, a feature which is stable, and thus has a small *α*_*i*_, and has a relatively large weight *w*_*i*_ is preferable.

By examining the learned step-sizes and weights $\bar{\frac{1}{\alpha }|w|}$ , we are finally able to separate the tracking and anticipatory touch predictions using an easily calculated metric (Figure 7(b)). As the step-sizes decrease, the value of both the tracking and anticipatory predictions rises; however, since the magnitude of the weight *w* is low for the bias bit, its weighted feature value remains low. This clarity in comparison carries over to the touch-left and touch-right predictions (Figure 7(b)). From Figure 6(b), we know that the tracking-based touch-left and touch-right predictions’ step-sizes never decay – the tracking predictions’ step-sizes maintain an average value of approximately 0.25 for the duration of the trials, while the anticipatory predictions’ step-sizes decay as the predictions are learnt. This results in a pronounced bifurcation between the two predictions. By looking at weighted features, we are able see and interpret what has been lost in our error estimates.

### 7.3. Final thoughts

The practice of using step-sizes to inform other aspects of learning is a well-established practice. For instance, learned step-sizes have been used for feature discovery (Mahmood & Sutton, 2013), and exploration methods (Linke et al., 2020). Recent work has suggested that step-sizes can be used to monitor the status of robots and indicate when physical damage has occurred in a system (Günther et al., 2019, 2020). Prior work in biological systems has consistently found there is more to representation learning than error minimization: for example, attention plays an important role in shaping how humans cognitively map their environment (Radulescu et al., 2019). This provides a suggestive interpretation of the benefits of adaptive step-sizes. Moreover, using internal learning measurements to evaluate predictive knowledge systems has been suggested in other works (Sherstan et al., 2016), although no existing applications of predictive knowledge use step-sizes for evaluation.

Using the learning method we generalized, AutoStep for GTD (*λ*), we can learn step-sizes online and incrementally as the agent is interacting with the environment. In situations where traditional prediction error metrics fail, the magnitude of learned weights and step-sizes enables differentiation between GVFs that are useful in informing further predictions, and GVFs which are not. In brief, we show that GVFs can be evaluated in a meaningful, scalable way using feature relevance.

## 8. Relevance and related work

In this manuscript, we focused our arguments on a particular set of predictions in two experiments; however, the conclusions drawn apply to real-world applications of GVFs as well. From industrial lazer welding (Günther et al., 2020) to autonomous vehicle navigation (Graves et al., 2021), error estimation is the means by which model quality is estimated prior to and during deployment. In situations like these where we evaluate models based on strict measures of accuracy, further decisions based on computed results are susceptible the evaluation and performance issues raised in this manuscript. While we focus on machine intelligence, similar observations about the primacy of prediction error have been made in cognitive neuroscience. For example, accuracy is not all that informs internal representations of location; additional factors such as attention also shape human spatial models (Radulescu et al., 2019). Moreover, attention has been used successfully to augment explainability when ML models are used for decision-making (Xu et al., 2015). We proposed that in general, solely considering the error a model is insufficient. While our discussion has focused in particular on applications of General Value Functions, we believe the conclusions drawn are not dependent on the learning methods themselves. The issues raised with respect to the use of models in decision-making transcend the learning methods discussed, and are relevant across all discussions of modelling in machine learning.

## 9. Conclusion

Agents often benefit from constructing general knowledge of their world. How the models that compose this knowledge are constructed and evaluated is a challenging open problem. In this paper, we critically discussed a common way of evaluating an agent’s knowledge: model accuracy with respect to observed values. As a first primary contribution of this work, we demonstrated how strict measures of accuracy can be misleading. We further showed how critical areas of performance can be hidden by biased measures of error, leading to a poor choice of model. Building on this observation, we next demonstrated how poor evaluation in learned models can lead to more serious errors in down-stream learning tasks (e.g. prediction) which depend on these models. As a final contribution, we proposed an alternative evaluation approach that instead examines an agent’s learned parameters as a basis for certifying learned knowledge, specifically focussing on learned weights and step-size values. Using these additional sources of information, we showed that we are able to differentiate between useful and useless model in a setting that was indistinguishable when using standard error or accuracy-based assessments. This paper therefore contributes a first look into how predictive models evaluation and use are related. Decoupling the evaluation of predictions from strict measures of accuracy is a key step towards building general, modular representations of knowledge.

## Notes

1 General Value Functions are a generalization of ordinary value functions: a central component of computational reinforcement learning. Value functions are used to estimate the value of a given state of the environment. General Value Functions are a generalization of traditional Value Functions to not just reward, but any signal accessible to the agent through its experience.

2 See (White, 2015) for an explanation of RUPEE on pages 119–122.

3 See Sutton and Barto (2019) for a discussion of eligibility traces and TD(*λ*).

4 This example is a simplification of the thought experiment introduced in Ring (2021).

5 For our function approximator, we use a tile coder. The tile coder outputs a binary feature vector – only a portion of all features are active on a given time-step. We multiple the average absolute step-size by the number of active features so that two function approximators with differing active feature sizes will have equivalent scale and can be compared.
